# PEDV inhibits HNRNPA3 expression by miR-218-5p to enhance cellular lipid accumulation and promote viral replication

**DOI:** 10.1128/mbio.03197-23

**Published:** 2024-01-23

**Authors:** Xiaojie Shi, Qi Zhang, Naling Yang, Quanqiong Wang, Yanxia Zhang, Xingang Xu

**Affiliations:** 1 College of Veterinary Medicine, Northwest A&F University, Yangling, Shaanxi, China; Virginia Polytechnic Institute and State University, Blacksburg, Virginia, USA; University of Illinois Urbana-Champaign, Urbana, Illinois, USA

**Keywords:** PEDV, host factors, HNRNPA3, lipid, viral replication complex

## Abstract

Porcine epidemic diarrhea virus (PEDV) requires complete dependence on the metabolic system of the host cell to complete its life cycle. There is a strong link between efficient viral replication and cellular lipid synthesis. However, the mechanism by which PEDV interacts with host cells to hijack cellular lipid metabolism to promote its replication remains unclear. In this study, PEDV infection significantly enhanced the expression of lipid synthesis-related genes and increased cellular lipid accumulation. Furthermore, using liquid chromatography-tandem mass spectrometry, we identified heterogeneous nuclear ribonucleoprotein A3 (HNRNPA3) as the interacting molecule of PEDV NSP9. We demonstrated that the expression of HNRNPA3 was downregulated by PEDV-induced miR-218-5p through targeting its 3′ untranslated region. Interestingly, knocking down HNRNPA3 facilitated the PEDV replication by promoting cellular lipid synthesis. We next found that the knockdown of HNRNPA3 potentiated the transcriptional activity of sterol regulatory element-binding transcription factor 1 (SREBF1) through zinc finger protein 135 (ZNF135) as well as PI3K/AKT and JNK signaling pathways. In summary, we propose a model in which PEDV downregulates HNRNPA3 expression to promote the expression and activation of SREBF1 and increase cellular lipid accumulation, providing a novel mechanism by which PEDV interacts with the host to utilize cellular lipid metabolism to promote its replication.

**IMPORTANCE:**

As the major components and structural basis of the viral replication complexes of positive-stranded RNA viruses, lipids play an essential role in viral replication. However, how PEDV manipulates host cell lipid metabolism to promote viral replication by interacting with cell proteins remains poorly understood. Here, we found that SREBF1 promotes cellular lipid synthesis, which is essential for PEDV replication. Moreover, HNRNPA3 negatively regulates SREBF1 activation and specifically reduces lipid accumulation, ultimately inhibiting PEDV dsRNA synthesis. Our study provides new insight into the mechanisms by which PEDV hijacks cell lipid metabolism to benefit viral replication, which can offer a potential target for therapeutics against PEDV infection.

## INTRODUCTION

Porcine epidemic diarrhea virus (PEDV), a member of the genus *Alphacoronavirus*, seriously restricts the development of the global pig industry (1). Lipids, a diverse group of biomolecules, play an indispensable role in cellular physiology and pathophysiology (2). Lipid droplets (LDs) are organelles that store neutral lipids and function in fatty acid transport and lipid signaling (3). Increasing data point that LDs also play a significant role in viral proliferation, highlighting the potential as therapeutic targets for viruses (4). Viruses can hijack the pathway of lipid biogenesis and exploit intracellular lipids to facilitate viral proliferation (5). Sterol regulatory element-binding transcription factor 1 (SREBF1) acts as the key transcription factor regulating cellular lipid metabolism, which regulates the synthesis of fatty acids by regulating the expression of a series of critical enzymes, such as fatty acid synthase (FASN) and acetyl-CoA carboxylase 1 (ACC1) (6, 7). Numerous studies have established that SREBF1-mediated lipid metabolism is the precondition for cellular lipid homeostasis and efficient viral replication (8–10). Nevertheless, whether PEDV infection affects cellular lipid synthesis to facilitate self-replication needs further exploration.

The genome of PEDV contains seven open reading frames (ORFs) encoding four structural proteins, 16 nonstructural proteins (NSPs), and ORF3 (11). Viral replication is a highly complex process, and it has been demonstrated that positive-strand RNA viruses can induce membrane rearrangements to facilitate viral replication complex (VRC) anchoring (12). These structures provide physical scaffolds for viral assembly and protect viral RNA from being recognized by cellular defense systems (13). Positive-stranded RNA viruses can utilize specific organelle membranes to form invaginated or protruding VRCs (14). Wherein the NSPs play an integral role in the formation of VRCs or virus-host interactions (15). Coxsackievirus B3 (CVB3) nonstructural 3A protein enhances PI4KIIIβ recruitment to membranes by binding to GBF1/Arf1, which in turn catalyzes the production of PI4P lipids to promote the biogenesis of lipid-rich organelles (16). Among the coronaviral NSPs, NSP9 is the conserved component of the VRC, and its dimerization is required for viral replication (17). Thus, NSP9 plays an essential part in the process of viral proliferation. Further exploration of the interaction between the host and NSP9 is the basis for revealing the replication mechanism of PEDV, which is meaningful for the prevention and treatment of PEDV.

The heterogeneous nuclear ribonucleoproteins (HNRNPs) family consists of 20 members that perform essential physiological functions such as RNA splicing, apoptosis, and translation regulation (18, 19). Moreover, the effective replication of viruses also requires the participation of the HNRNP family proteins. For example, HNRNP K supports vesicular stomatitis virus infection by inhibiting apoptosis and suppressing antiviral protein expression (20). As a member of the HNRNP family, studies on heterogeneous nuclear ribonucleoprotein A3 (HNRNPA3) are relatively scarce. Most of the current studies on HNRNPA3 are directed at its function in cancer fields, but the role of HNRNPA3 in virus replication has not been reported.

In this study, we identified the importance of SREBF1 activation and lipid accumulation for PEDV replication and indicated HNRNPA3 as a negative regulator of lipid synthesis during PEDV infection. In summary, our data highlight the antiviral effect of HNRNPA3, which proposes a new approach to the prevention of PEDV infection.

## RESULTS

### PEDV induces lipid accumulation through the activation of SREBF1

**Fig 1:**
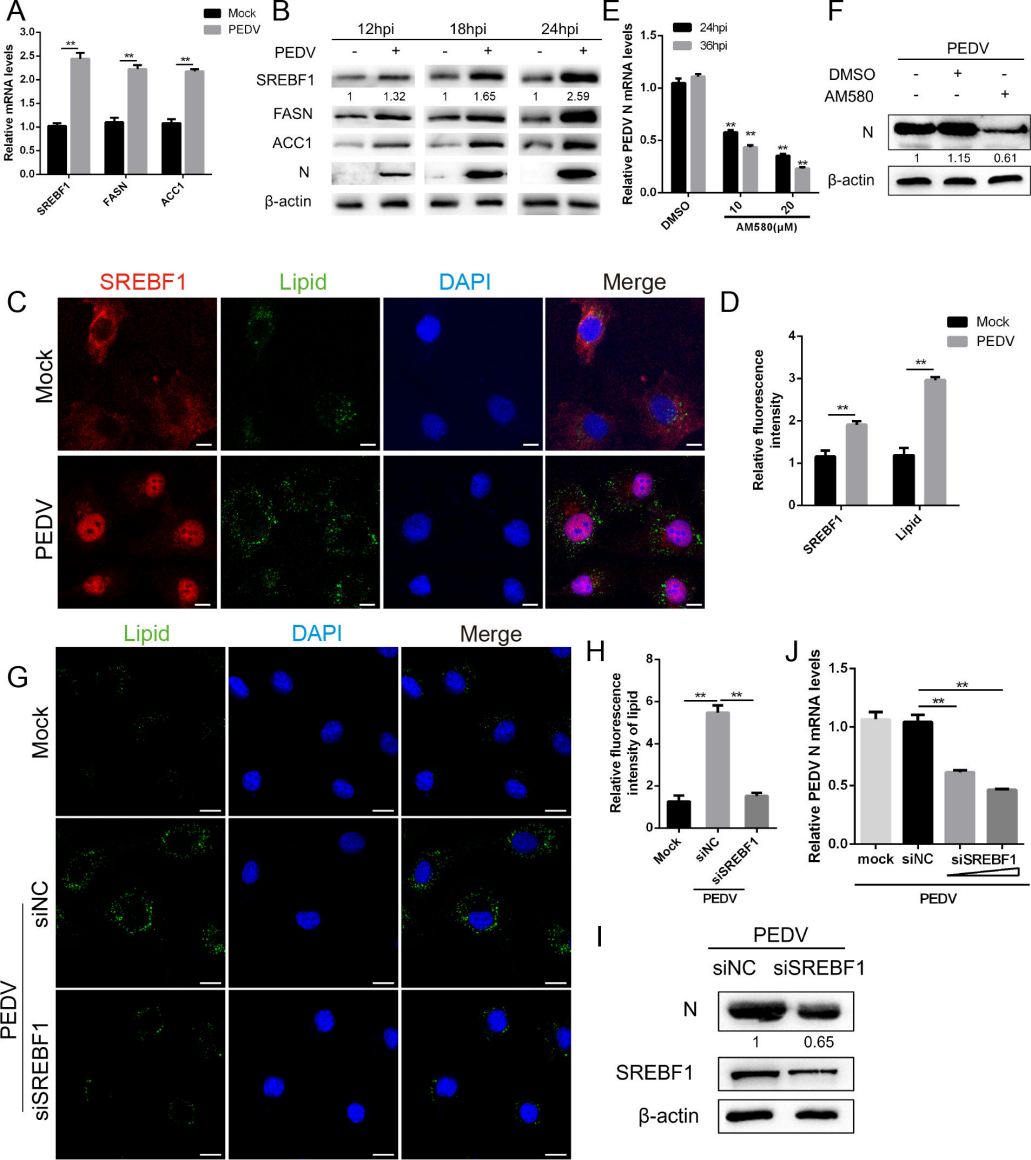
PEDV induces lipid accumulation through the activation of SREBF1. (**A**) Marc-145 cells were either mock-infected or infected with PEDV (multiplicity of infection [MOI] = 1) for 24 h, and the mRNA levels of SREBF1, FASN, and ACC1 were determined by quantitative PCR (qPCR). (**B**) Marc-145 cells were infected with PEDV (MOI = 1) for 12, 18, and 24 h. The protein levels of SREBF1, FASN, and ACC1 were detected by western blot. β-Actin was used as a loading control. (**C and D**) Marc-145 cells were infected with PEDV (MOI = 1) for 24 h. The production and subcellular localization of SREBF1 were detected by immunofluorescence assay. Scale bar, 8 µm. The fluorescence intensity of SREBF1 was analyzed with ImageJ. (**E**) Marc-145 cells were infected with PEDV (MOI = 1) and treated with different concentrations of AM580 (10 and 20 µM) or vehicle (dimethyl sulfoxide [DMSO], 1:1,000), and the mRNA levels of PEDV-N were quantified by qPCR. (**F**) Marc-145 cells were infected with PEDV (MOI = 1) and treated with AM580 (10 µM) or vehicle (DMSO, 1:1,000). The protein levels of PEDV-N were detected by western blot. β-Actin was used as a loading control. (**G and H**) Marc-145 cells were transfected with siSREBF1 or siNC and then infected with PEDV (MOI = 1) for 24 h. The contents of cellular lipid droplets (green) and the nuclei (blue) were detected by immunofluorescence assay. Scale bar, 10 µm. The fluorescence intensity of lipids was analyzed with ImageJ. (**I and J**) Marc-145 cells were transfected with siSREBF1 or siNC and then infected with PEDV (MOI = 1) for 24 h. The mRNA and protein levels of SREBF1 and PEDV-N were detected by qPCR and western blot. Three independent experiments were carried out. Error bars represent the mean ± SD for triplicate experiments. **P < 0.01.

Cellular lipids can participate in several processes of viral proliferation, such as attachment, viral genome replication, and viral budding (21). SREBF1 is a primary regulator of cellular lipid synthesis and lipid homeostasis (22). To investigate whether PEDV infection influences the expression of SREBF1, the PEDV-infected cells were harvested at various time points for further analysis. The mRNA levels of SREBF1 and its downstream genes (*FASN* and *ACC1*) were increased approximately twofold at 24 hpi (Fig. 1A). Furthermore, a significant elevation of SREBF1 protein expression was detected in a time-dependent manner and kept at a comparatively elevated level (Fig. 1B). To validate the effect of PEDV on the cellular localization of SREBF1, mock-infected and PEDV-infected cells were detected by immunofluorescence. Compared with the control, nuclear translocation of SREBF1 and increased accumulation of cellular lipids were observed in PEDV-infected cells (Fig. 1C and D). It is widely recognized that lipid droplets are composed of neutral lipids such as triglycerides and sterol esters (23). Thus, we examined the changes in cellular triglycerides at various intervals after PEDV infection. The results showed a gradual increase in triglyceride content after infection, peaking at 24 hpi (Fig. S1A).

Moreover, cells were treated with AM580, an inhibitor of SREBF1, followed by PEDV infection. After treating cells with AM580, PEDV N mRNA (significantly, in a dose-dependent manner) and protein levels were decreased (Fig. 1E and F). Meanwhile, the immunofluorescence assay results displayed that the content of lipids in AM580-treated cells was lower than that in the control group (Fig. S1B and C). Since we confirmed that PEDV infection induces SREBF1 activation, we further investigated the role of SREBF1 in PEDV infection. For this purpose, siRNAs against SREBF1 were transfected into cells and then incubated with PEDV. Lipid accumulation in siSREBF1-transfected cells was attenuated by immunofluorescence assays (Fig. 1G and H). Additionally, the western blot results indicated that the knockdown of SREBF1 resulted in significant inhibition of PEDV replication (Fig. 1I and J). These data indicate that PEDV infection increases the expression of SREBF1, while SREBF1 may promote lipid accumulation, thereby favoring PEDV replication.

### HNRNPA3 is downregulated by PEDV infection and impairs the replication of PEDV

**Fig 2:**
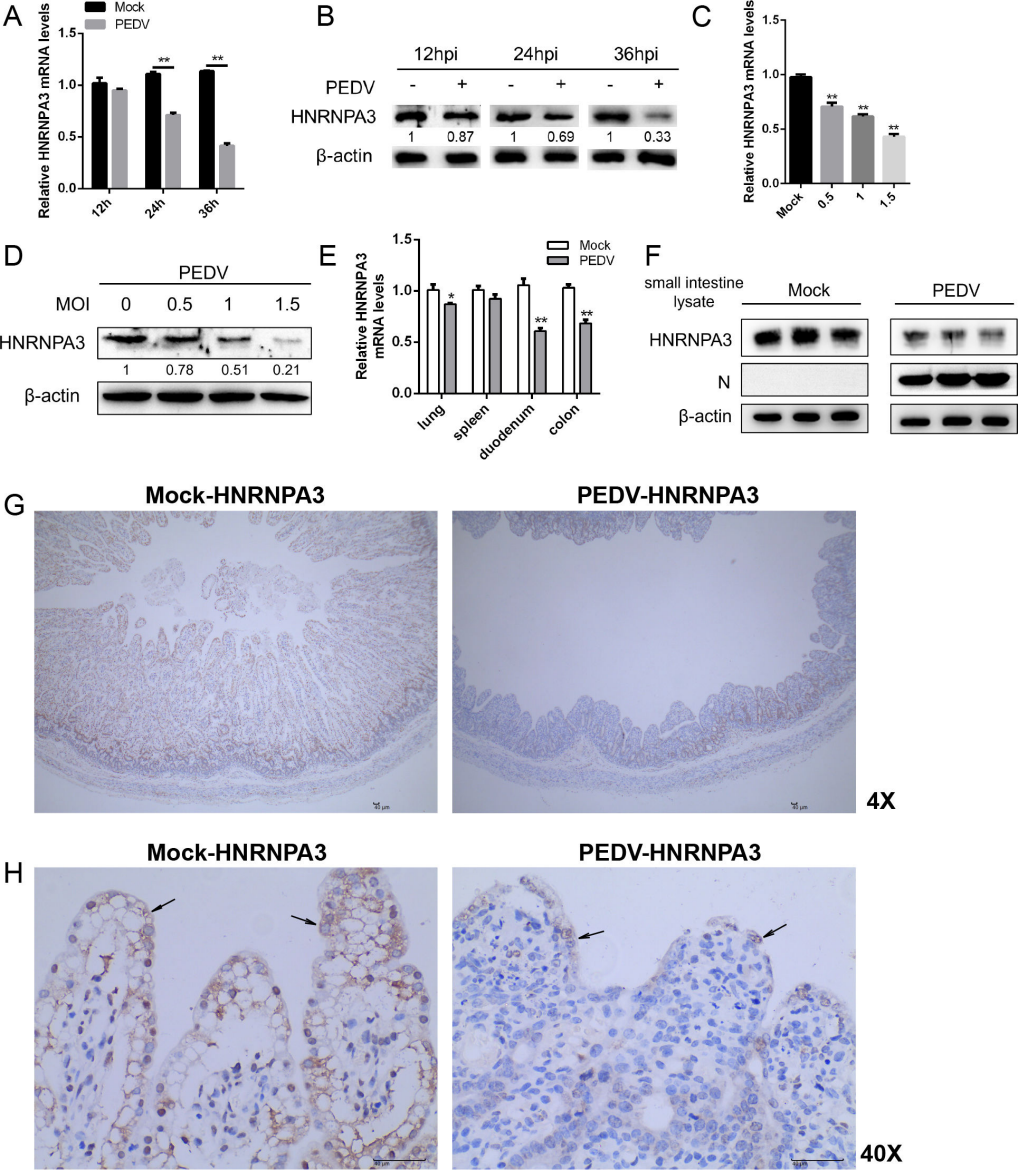
HNRNPA3 is downregulated by PEDV infection. (**A and B**) Marc-145 cells were either mock-infected or infected with PEDV (MOI = 1) for 12, 24, and 36 h. The mRNA and protein levels of HNRNPA3 were determined by qPCR and western blot. β-Actin was used as a loading control. (**C and D**) Marc-145 cells were either mock-infected or infected with PEDV (MOI = 0.5, 1.0, 1.5) for 24 h. The mRNA and protein levels of HNRNPA3 were determined by qPCR and western blot. β-Actin was used as a loading control. (**E and F**) The mRNA and protein levels of HNRNPA3 in the tissue samples of the mock-infected and PEDV-infected groups were determined by qPCR and western blot. β-Actin was used as a loading control. (**G and H**) Immunohistochemistry staining was used to analyze the expression of HNRNPA3 in mock-infected and PEDV-infected groups (magnification: ×4 and ×40). HNRNPA3 was stained deep yellow-brown (black arrows). Three independent experiments were carried out. Error bars represent the mean ± SD for triplicate experiments. *P < 0.05, **P < 0.01.

In the previous experiments, we discovered that cellular lipids are essential for the efficient replication of PEDV. It is well known that VRC formation requires the involvement of lipids, and NSP9 is a component of VRC (24). Therefore, we performed mass spectrometry to screen the cell proteins interacting with PEDV NSP9 to realize the mechanisms of PEDV-host interactions (Fig. S2A). Next, we confirmed that the mRNA and protein levels of HNRNPA3 were markedly decreased in a time-dependent (Fig. 2A and B) and dose-dependent relationship during PEDV infection (Fig. 2C and D). To further validate the expression levels of HNRNPA3 *in vivo*, the newborn piglets in the PEDV-infected group were infected by oral inoculation. Watery diarrhea and vomiting symptoms were observed in piglets at 3 dpi, and then all piglets were anesthetized and euthanized. The tissue samples were collected, and we found that HNRNPA3 expression in the PEDV-infected group was lower than that in the mock-infected group (Fig. 2E and F). Immunohistochemistry was employed to identify whether protein levels of HNRNPA3 in small intestinal villi were changed due to PEDV infection. In agreement with our *in vitro* findings, the *in vivo* results suggested that HNRNPA3 expression was considerably reduced in PEDV-infected piglets (Fig. 2G and H; Fig. S2B). The above results reveal that the expression levels of HNRNPA3 were substantially reduced *in vivo* and *in vitro* during PEDV infection.

**Fig 3:**
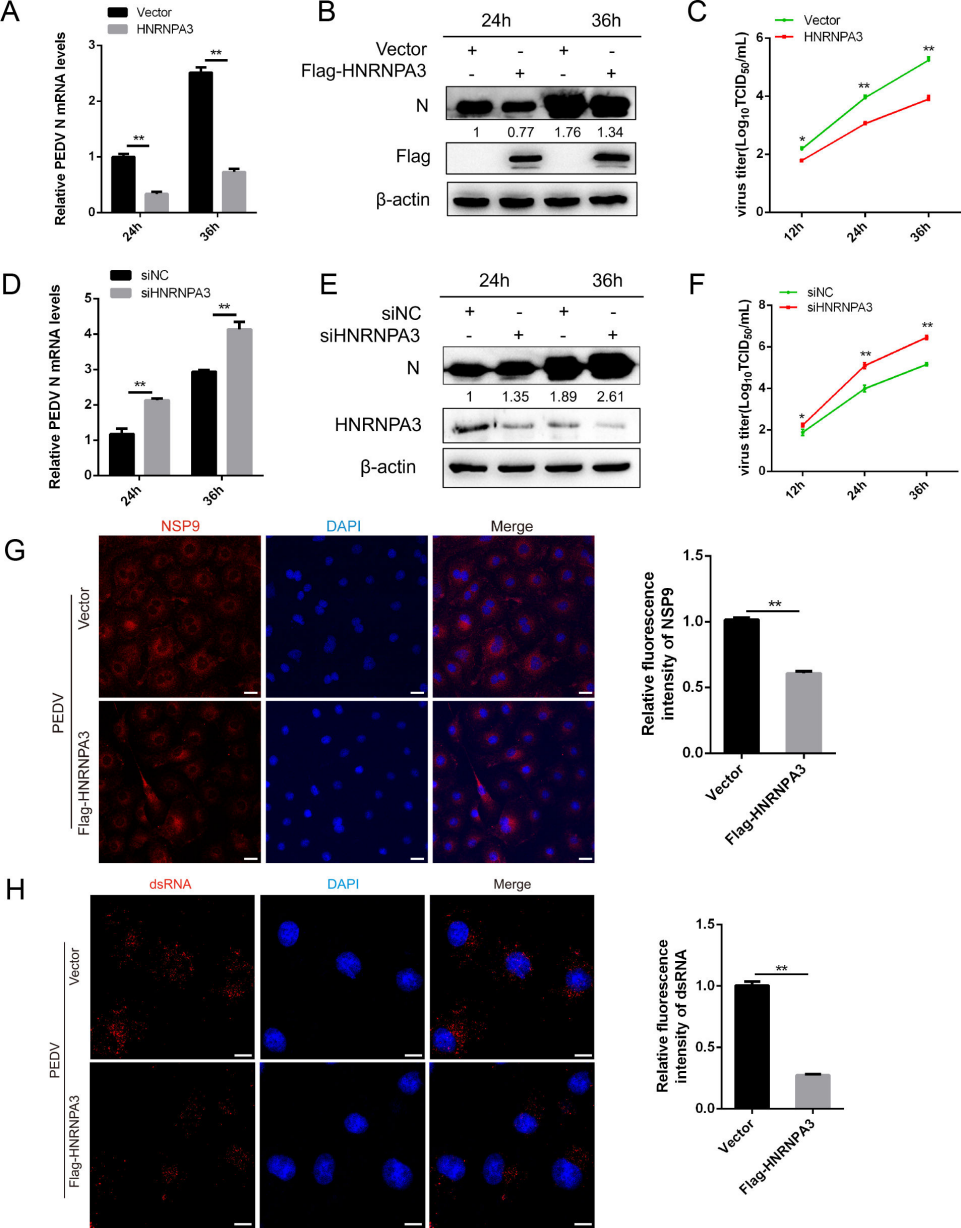
HNRNPA3 impairs the replication of PEDV. (**A and B**) Marc-145 cells were transfected with vector or Flag-HNRNPA3 and infected with PEDV (MOI = 1) for 24 and 36 h. The mRNA and protein levels of PEDV-N were determined by qPCR and western blot. β-Actin was used as a loading control. (**C**) Marc-145 cells were treated as described above, and the supernatant was harvested to assess virus titer by 50% tissue culture infective dose (TCID_50_). (**D and E**) Marc-145 cells were transfected with siNC or siHNRNPA3 and infected with PEDV (MOI = 1) for 24 and 36 h. The mRNA and protein levels of PEDV-N were determined by qPCR and western blot. β-Actin was used as a loading control. (**F**) Marc-145 cells were treated as described above, and the supernatant was harvested to assess virus titer by TCID_50_. (**G**) Marc-145 cells were transfected with vector or Flag-HNRNPA3 and infected with PEDV (MOI = 1) for 24 h. The expression of NSP9 was detected by immunofluorescence assay. Scale bar, 20 µm. The fluorescence intensity of NSP9 was analyzed with ImageJ. (**H**) Marc-145 cells were transfected with vector or Flag-HNRNPA3 and infected with PEDV (MOI = 1) for 12 h. The content of PEDV dsRNA (red) was detected by immunofluorescence assay. Scale bar, 10 µm. The fluorescence intensity of dsRNA was analyzed with ImageJ. Three independent experiments were carried out. Error bars represent the mean ± SD for triplicate experiments. *P < 0.05, **P < 0.01.

To explore whether HNRNPA3 affects PEDV replication, Marc-145 cells were transfected with empty vectors or Flag-HNRNPA3 plasmids and infected with PEDV. We observed that the overexpression of HNRNPA3 induces a substantial decrease in the transcript and protein levels of PEDV N (Fig. 3A and B). Similarly, the virus titers in the Flag-HNRNPA3-transfected cells were markedly lower than those in the vector-transfected cells (Fig. 3C). To substantiate the action of HNRNPA3 in viral replication, the specific siRNAs against HNRNPA3 were designed for further assay, and the interference efficiency was performed by quantitative PCR (qPCR) and western blot (Fig. S3A and B). Knocking down HNRNPA3 significantly increased the expression of PEDV N; moreover, the virus titers were also higher than those in siNC-transfected cells (Fig. 3D through F). Immunofluorescence assay results confirmed that overexpression of HNRNPA3 suppressed PEDV replication (Fig. 3G). To investigate whether the early steps of the PEDV life cycle were affected by HNRNPA3, we performed the attachment and entry assay. As shown in Fig. S4, HNRNPA3 had no significant effects on PEDV attachment and entry. To further examine whether HNRNPA3 affects PEDV replication, cells were transfected with Flag-HNRNPA3 plasmids and infected with PEDV and then stained for dsRNA, as a marker of viral replication (Fig. 3H). The levels of PEDV dsRNA in HNRNPA3-transfected cells were lower than those in the control groups (Fig. 3H). Overall, the results indicate that HNRNPA3 impairs PEDV replication through affecting the synthesis of dsRNA.

### PEDV-induced miR-218-5p negatively regulates the expression of HNRNPA3

**Fig 4:**
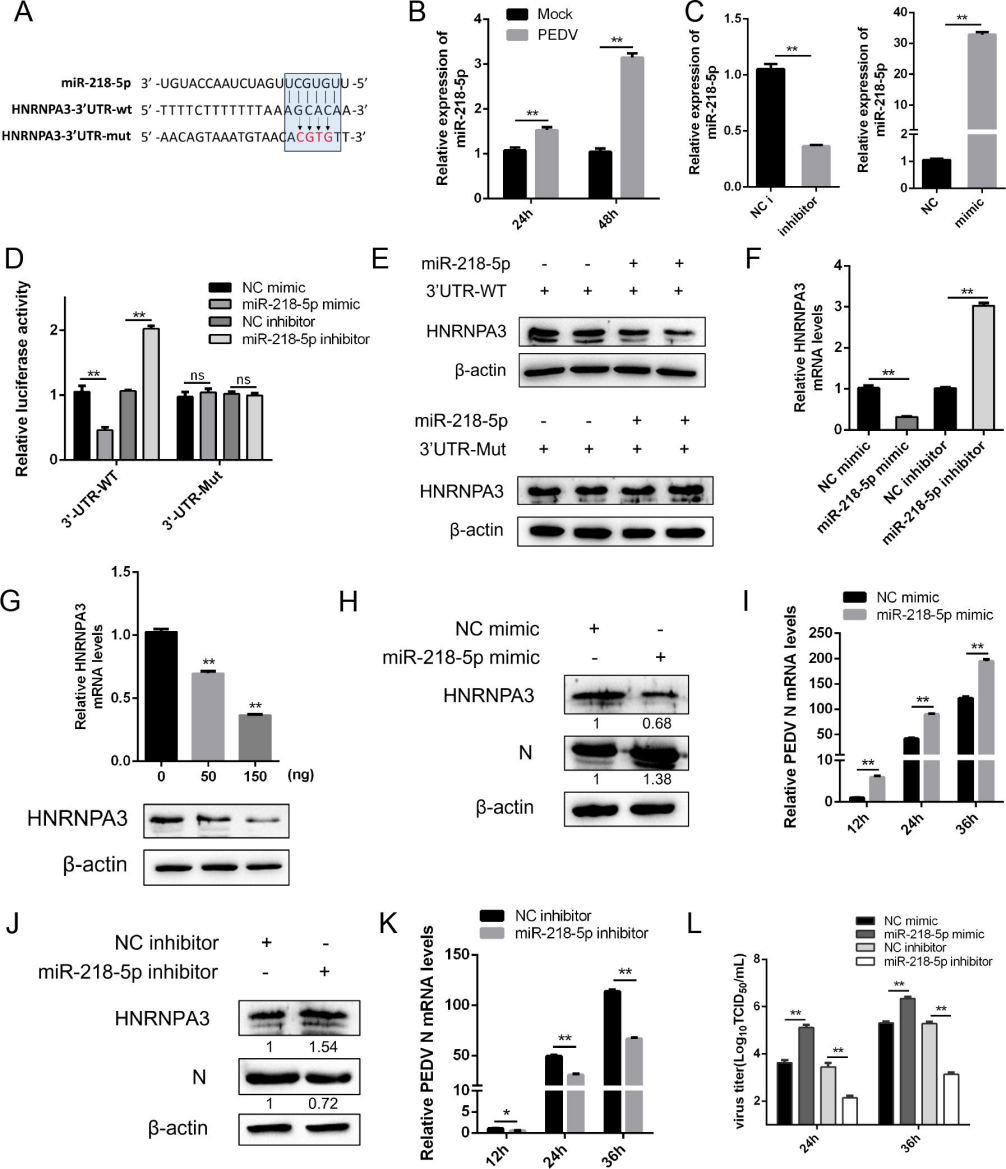
PEDV-induced miR-218-5p negatively regulates the expression of HNRNPA3. (**A**) Predicted miR-218-5p binding sites in the 3′-UTR of the HNRNPA3 mRNA. Lines indicate the matches in seed regions, and the mutations (indicated by arrows) were generated in the binding sites. (**B**) Marc-145 cells were infected with PEDV (MOI = 1) for 24 and 48 h, and the expression of miR-218-5p was detected by qPCR. (**C**) Marc-145 cells were transfected with miR-218-5p mimic or inhibitor, and the expression of miR-218-5p was detected by qPCR. (**D**) Marc-145 cells were transfected with the wild type or mutant of the HNRNPA3 3′-UTR reporter plasmids and miR-218-5p mimic or negative control (NC) mimic and miR-218-5p inhibitor or NC inhibitor for 36 h before the luciferase assays. (**E**) Marc-145 cells were co-transfected with miR-218-5p mimic and HNRNPA3 3′-UTR-WT or HNRNPA3 3′-UTR-Mut for 36 h and were subjected to western blot. (**F**) Marc-145 cells were transfected with miR-218-5p mimic or NC mimic and miR-218-5p inhibitor or NC inhibitor for 36 h, and the mRNA levels of HNRNPA3 were detected by qPCR. (**G**) Marc-145 cells were transfected with an increasing amount of miR-218-5p mimic, and the expression of HNRNPA3 was detected by qPCR and western blot. (**H and J**) Marc-145 cells were transfected with miR-218-5p mimic or NC mimic and miR-218-5p inhibitor or NC inhibitor and infected with PEDV (MOI = 0.5) for 24 h. The protein levels of HNRNPA3 and PDV-N were detected by western blot. β-Actin was used as a loading control. (**I and K**) Marc-145 cells were transfected with miR-218-5p mimic or NC mimic and miR-218-5p inhibitor or NC inhibitor and infected with PEDV (MOI = 0.5) for 12, 24, and 36 h. The mRNA levels of PEDV-N were detected by qPCR. (**L**) Marc-145 cells were transfected with NC mimic, miR-218-5p mimic, NC inhibitor, or miR-218-5p inhibitor for 24 h and infected with PEDV (MOI = 0.5), and the supernatant was harvested to assess virus titer by TCID_50_. Three independent experiments were carried out. Error bars represent the mean ± SD for triplicate experiments. *P < 0.05, **P < 0.01. ns, not significant.

Our previous studies found that many miRNAs were significantly upregulated or downregulated after PEDV infection (25). Therefore, we explored whether there was an endogenous miRNA that specifically inhibited HNRNPA3 expression. We screened out miR-218-5p, which targets the 3′ untranslated region (3′-UTR) of HNRNPA3 mRNA through TargetScan and miRanda software, and miR-218-5p expression was raised by PEDV infection (Fig. 4A and B). As shown in Fig. 4C, the transfection of miR-218-5p inhibitor significantly reduced miR-218-5p expression, while miR-218-5p mimic increased miR-218-5p expression. The miR-218-5p mimics sharply decreased the luciferase activity in HNRNPA3 3′-UTR-wild type (WT), however, with mutation of the HNRNPA3 3′-UTR, the inhibitory effects of miR-218-5p were abolished (Fig. 4D). Additionally, miR-218-5p mimics impaired HNRNPA3 expression in 3′-UTR-WT-transfected cells, whereas it had no effect on 3′-UTR-Mut-transfected cells (Fig. 4E).

To verify whether miR-218-5p regulates HNRNPA3 expression, we detected the expression level of HNRNPA3 in the cells transfected with miR-218-5p mimic. Whether at the mRNA or protein level, the miR-218-5p mimic showed an inhibitory effect on the expression of HNRNPA3 (Fig. 4F and G). In contrast, miR-218-5p inhibitor markedly augmented HNRNPA3 expression (Fig. 4F). Given that HNRNPA3 inhibits PEDV replication, we determined whether miR-218-5p mimic and inhibitor affect PEDV replication. The results disclosed that the expression of PEDV N was markedly affected by miR-218-5p (Fig. 4H through K). Consistently, miR-218-5p significantly impaired PEDV replication through TCID_50_ assay (Fig. 4L). Together, our findings indicate that miR-218-5p downregulates HNRNPA3 expression by binding to the 3′-UTR, hence affecting PEDV replication.

### HNRNPA3 inhibits SREBF1 expression and lipid accumulation during PEDV infection

**Fig 5:**
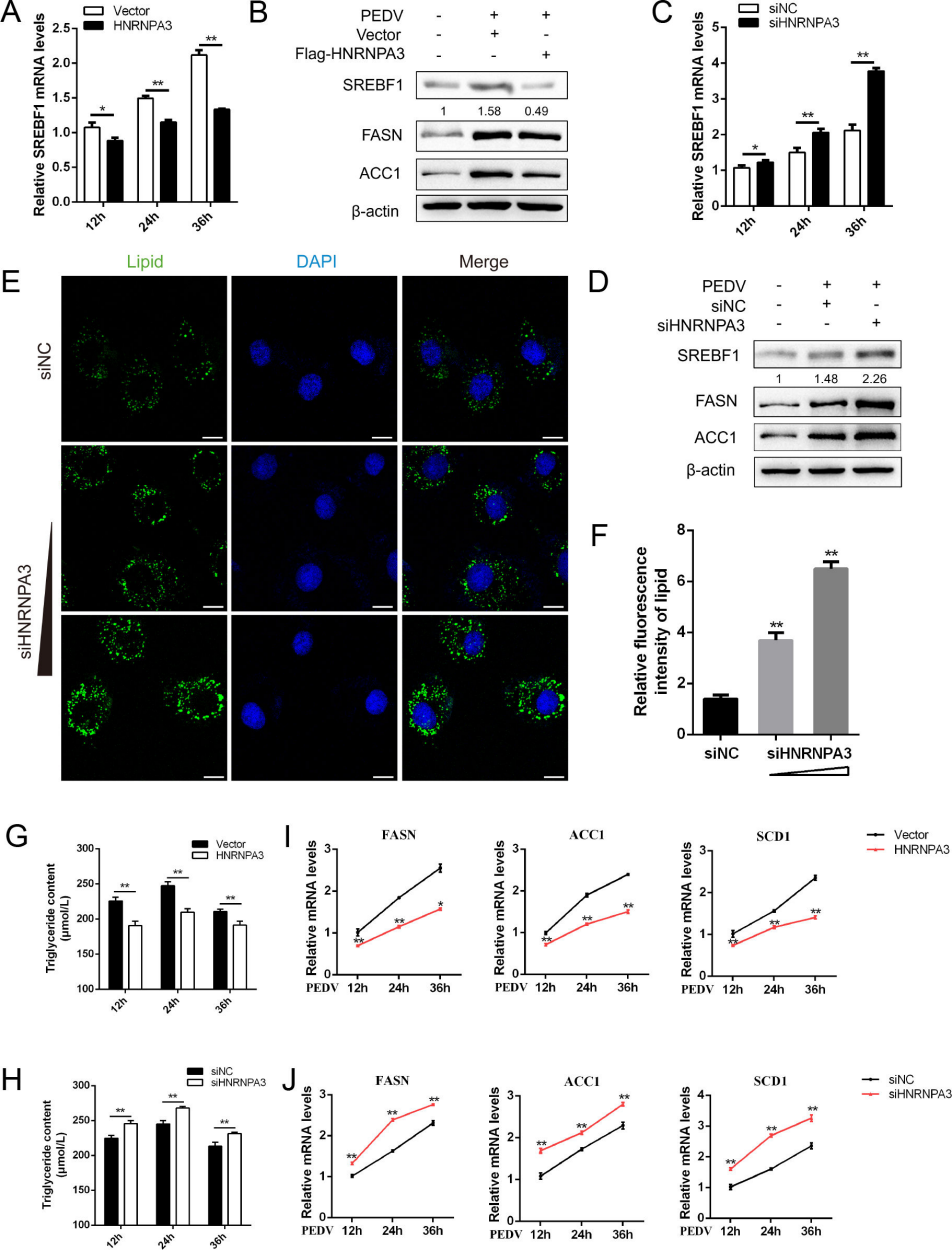
HNRNPA3 inhibits SREBF1 expression and lipid accumulation during PEDV infection. (**A and C**) Marc-145 cells were transfected with vector or Flag-HNRNPA3 and siNC or siHNRNPA3 and infected with PEDV (MOI = 0.5) for 12, 24, and 36 h. The mRNA levels of SREBF1 were determined by qPCR. (**B and D**) Marc-145 cells were transfected with vector or Flag-HNRNPA3 and siNC or siHNRNPA3 and infected with PEDV (MOI = 0.5) for 24 h. The protein levels of SREBF1 were determined by western blot. β-Actin was used as a loading control. (**E and F**) Marc-145 cells were transfected with siNC or increasing amounts of siHNRNPA3 and then infected with PEDV (MOI = 1). The content of cellular lipid droplets (green) and the nuclei (blue) were detected by immunofluorescence assay. Scale bar, 10 µm. The fluorescence intensity of lipids was analyzed with ImageJ. (**G and H**) Marc-145 cells were transfected with vectors or Flag-HNRNPA3 and siNC or siHNRNPA3, and infected with PEDV (MOI = 0.5). The triglyceride contents were detected. (**I and J**) Marc-145 cells were transfected with vector or Flag-HNRNPA3 and siNC or siHNRNPA3 and then infected with PEDV (MOI = 0.5) for 24 h. The mRNA levels of FASN, ACC1, and SCD1 were determined by qPCR. Three independent experiments were carried out. Error bars represent the mean ± SD for triplicate experiments, *P < 0.05, **P < 0.01.

Next, we intended to investigate whether HNRNPA3 affects lipid accumulation during PEDV infection. A significant time-dependent decrease in SREBF1 mRNA expression was observed after transfection with Flag-HNRNPA3 plasmids (Fig. 5A). Additionally, overexpression of HNRNPA3 reduced the production of SREBF1 as well as its downstream genes FASN and ACC1 (Fig. 5B). Meanwhile, SREBF1 mRNA and protein levels in siHNRNPA3-transfected cells were increased compared to the control (Fig. 5C and D). We performed immunofluorescence assay to further examine the effect of HNRNPA3 on lipid accumulation. A noticeable increase in lipid accumulation was detected in a dose-dependent manner in siHNRNPA3-transfected cells, further validated by fluorescence intensity analysis (Fig. 5E and F). In addition, a marked increase in the triglyceride content was detected in siHNRNPA3-transfected cells, and overexpression of HNRNPA3 reduced the triglyceride content (Fig. 5G and H; Fig. S5A). Next, we examined the impacts of HNRNPA3 on the mRNA levels of FASN, ACC1, and SCD1. As shown in Fig. 5I, the mRNA abundance of these genes was downregulated in Flag-HNRNPA3 plasmid-transfected cells. In contrast, the mRNA levels of FASN, ACC1, and SCD1 in siHNRNPA3-cells were upregulated relative to the control (Fig. 5J). Collectively, these results indicate that HNRNPA3 negatively regulates the expression of SREBF1 and its downstream genes, as well as cellular lipid accumulation during PEDV infection.

### Role of ZNF135 transcription factor in SREBF1 activation by HNRNPA3

**Fig 6:**
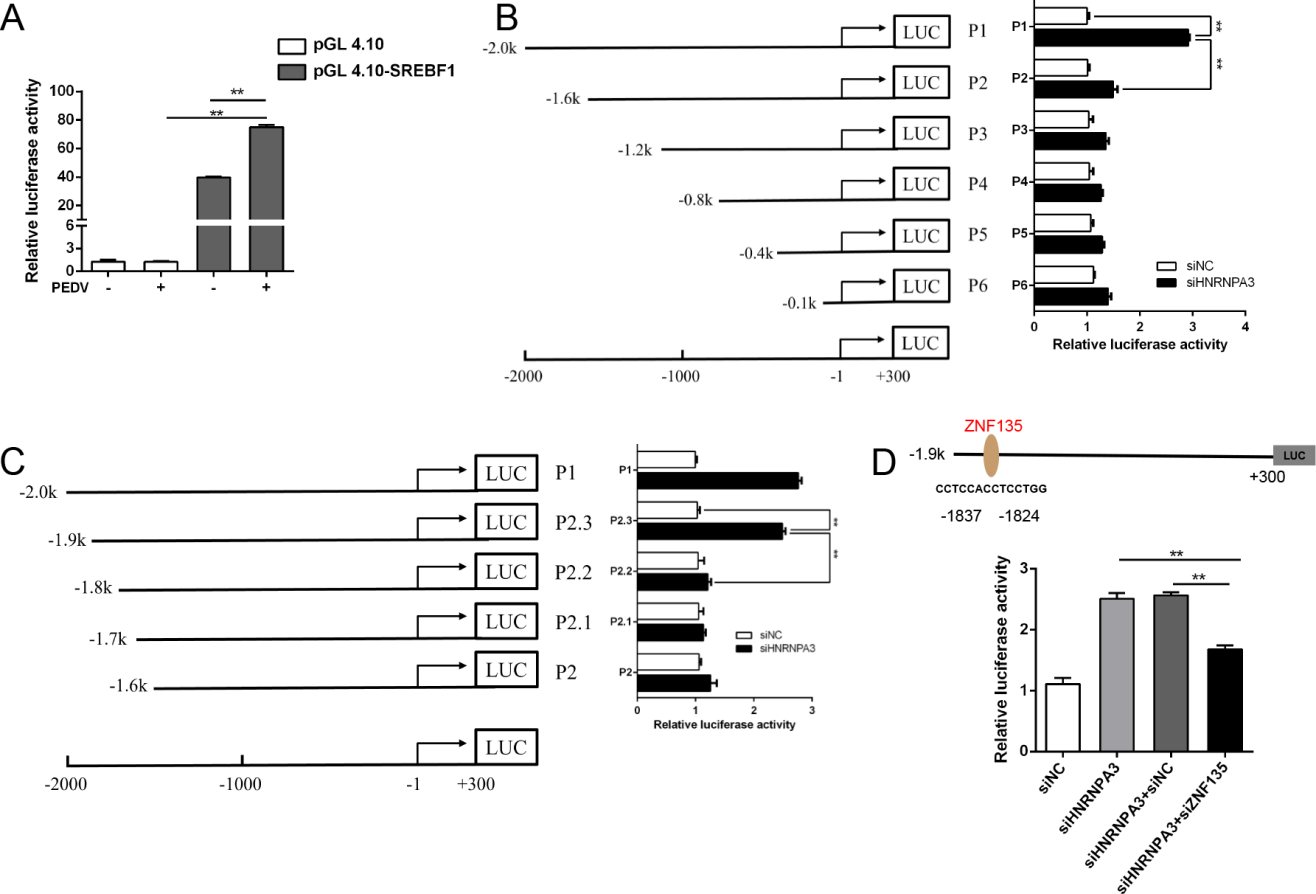
Role of ZNF135 transcription factor in SREBF1 activation by HNRNPA3. (**A**) Marc-145 cells were transfected with the pGL4.10-SREBF1 promoter and phRL-TK for 24 h and infected with PEDV (MOI = 0.5) for 24 h. The cells were harvested for dual-luciferase assays, and the relative luciferase activity was set to 1. (**B and C**) Marc-145 cells were transfected with siHNRNPA3 and a different region of SREBF1 promoter-reporter plasmids. The cells were harvested for dual-luciferase assays. (**D**) Regulatory elements in the SREBF1 promoter region were predicted with JASPAR (http://jaspar.genereg.net/). pGL4.10-SREBF1-promoter was co-transfected with siHNRNPA3 and/or ZNF135-specific siRNAs into Marc-145 cells. The cells were harvested for dual-luciferase assays, and the relative luciferase activity was set to 1. Three independent experiments were carried out. Error bars represent the mean ± SD for triplicate experiments. *P < 0.05, **P < 0.01.

To detect the key SREBF1 promoter regions for its activation by HNRNPA3, six regions (−2,000/300, –1,600/300, −1,200/300, –800/300, −400/300, and −100/300, designated P1–P6) were cloned into the pGL4.10 vector to construct pGL4.10-SREBF1 promoter plasmids. The luciferase assay results suggested that PEDV infection enhanced SREBF1 promoter activity (Fig. 6A). The promoter activity decreased significantly due to the deletion from −2,000 to −1,600, demonstrating the presence of essential factors in this region (Fig. 6B). The promoter region (−2,000 to −1,600) was further truncated to determine the minimum range of the promoter. We ultimately identified that the region between −1,900 and −1,800 was required for SREBF1 activation by HNRNPA3 (Fig. 6C). Sequence analysis with JASPAR revealed that the −1,900 to −1,800 region contains the binding site for the transcription factor ZNF135 (Fig. 6D). To explore the role of ZNF135 in regulating SREBF1 promoter activity, specific siRNAs were used to downregulate ZNF135 expression. The dual luciferase assay demonstrated that the knockdown of HNRNPA3 upregulates SREBF1 promoter activity, which is in concordance with the previous results (Fig. 6D). The control siRNA did not affect SREBF1 promoter activity, whereas the knockdown of ZNF135 inhibited the activity induced by the knockdown of HNRNPA3 (Fig. 6D). Overall, these data demonstrate that ZNF135 binds to the SREBF1 promoter and plays a function in SREBF1 activation.

### PI3K/AKT and JNK mediate the effect of HNRNPA3 on SREBF1 activation

**Fig 7:**
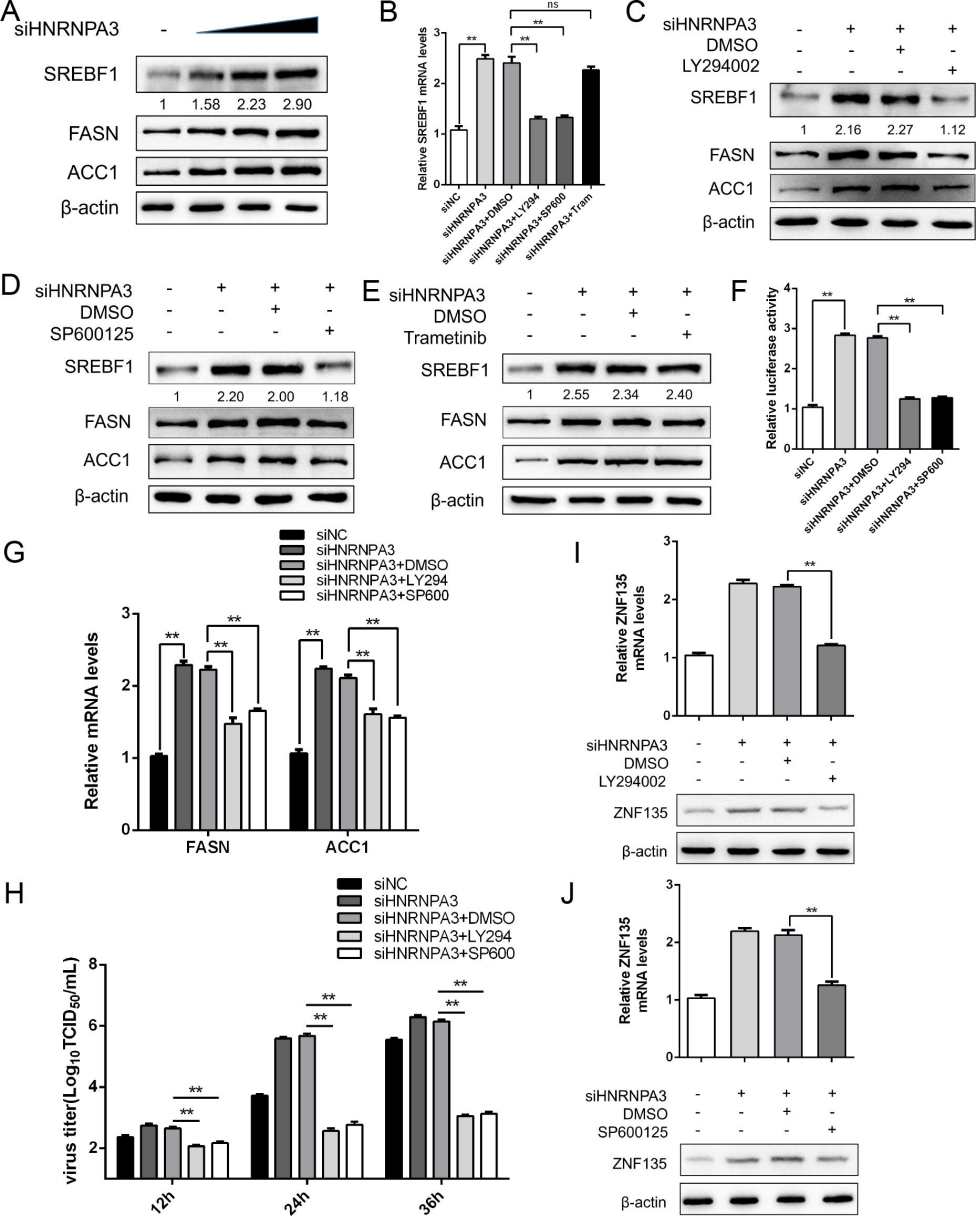
PI3K/AKT and JNK mediate the effect of HNRNPA3 on SREBF1 activation. (**A**) Marc-145 cells were transfected with siNC or increasing amounts of siHNRNPA3 and infected with PEDV (MOI = 0.5) for 24 h. The protein levels of SREBF1, FASN, and ACC1 were determined by western blot. β-Actin was used as a loading control. (**B**) Marc-145 cells were transfected with siHNRNPA3 and infected with PEDV (MOI = 0.5), then incubated with LY294002 (LY294, 10 µM), SP600125 (SP600, 10 µM), trametinib (Tram, 10 µM), or DMSO (1:1,000). The mRNA levels of SREBF1 were determined by qPCR. (**C–E**) Marc-145 cells were transfected with siHNRNPA3 and infected with PEDV (MOI = 0.5), then incubated with LY294002/SP600125/trametinib or DMSO. The protein levels of SREBF1 were determined by western blot. β-Actin was used as a loading control. (**F**) Marc-145 cells were transfected and treated as described above. The cells were harvested for dual-luciferase assays. (**G and H**) Marc-145 cells were transfected and treated as described above. The mRNA levels of FASN and ACC1 were determined by qPCR, and the supernatant was harvested to assess the virus titer by TCID_50_. (**I and J**) Marc-145 cells were transfected and treated as described above. The mRNA and protein levels of ZNF135 were determined by qPCR and western blot. β-actin was used as a loading control. Three independent experiments were carried out. Error bars represent the mean ± SD for triplicate experiments. *P < 0.05, **P < 0.01. ns, not significant.

Previous studies have indicated that the PI3K/Akt and MAPK cascade signaling pathways participate in SREBP1 activation (26, 27). First, we found that the siRNAs of HNRNPA3 promoted the expression of SREBF1 in a dose-dependent manner during PEDV infection (Fig. 7A). To identify the specific signaling pathways participating in HNRNPA3-mediated activation of SREBF1, the Marc-145 cells were treated with small molecule inhibitors that targeted PI3K (LY294002), JNK (SP600125), and MEK (trametinib), respectively. The cell viability results showed that the inhibitors did not affect cell viability at the concentrations used in this study (Fig. S5B).

LY294002 and SP600125 suppressed the upregulation of SREBF1 mRNA promoted by siHNRNPA3, while trametinib had no significant effect on SREBF1 mRNA regulation (Fig. 7B). Similar findings were verified in the results of the western blot assay. siHNRNPA3 cells were infected with PEDV and then treated with different inhibitors. Compared with the control group, SREBF1 expression in LY294002-treated cells and SP600125-treated cells was inhibited to some extent (Fig. 7C and D). Contrarily, there was no significant difference in the expression of SREBF1 in trametinib-treated cells (Fig. 7E). Additionally, the dual-luciferase reporter assays indicated that the activity of SREBF1 was impaired in LY294002-treated cells and SP600125-treated cells (Fig. 7F). As expected, the mRNA levels of FASN and ACC1 and the virus titers were decreased under the treatment of LY294002 and SP600125 (Fig. 7G and H). Additionally, specific siRNAs targeting PI3K and JNK were used to validate the role of these pathways in regulating SREBF1 expression by HNRNPA3. The expression levels of PI3K and JNK were significantly reduced (Fig. S6A and B). Meanwhile, the cells transfected with siPI3K and siJNK could decrease the siHNRNPA3-mediated increase in SREBF1 protein expression (Fig. S6C and D). Furthermore, the expression of transcription factor ZNF135 in LY294002-treated or SP600125-treated groups was lower than that in control groups (Fig. 7I and J). These data indicate that PI3K/AKT and JNK pathways are essential in HNRNPA3-mediated SREBF1 activation.

## DISCUSSION

During viral infection, the virus interacts with host cellular factors to create conditions for its efficient replication. PEDV is a worldwide-distributed porcine enteropathogenic coronavirus; however, there is still a lack of understanding about PEDV-host interactions. Cellular lipids, as important biomolecules in cells, are also essential in the life cycle of viruses (28). Many studies in recent years have reported that viruses could hijack cellular lipid biogenesis to provide the substances needed for virus replication. For example, DENV infection resulted in the localization of FASN to the viral replication compartment and increased fatty acid synthesis (29). Additionally, the expression of genes related to cholesterol regulation such as SREBP2 and FXR was upregulated during PEDV infection (30). Here, we found increased expression of SREBF1 as well as increased lipid accumulation during PEDV infection, which may favor viral replication. The knockdown of SREBF1 suppressed PEDV replication by reducing cellular lipid content, which is consistent with the effects of SREBF1 activation in another study (31). Inhibition of SREBF1 activation using 25-hydroxycholesterol or AM580 also resulted in infection suppression by various viruses (32–35). A similar observation was found in our study, where PEDV replication was notably inhibited after AM580 treatment. Moreover, the knockdown of SREBF1 suppressed PEDV replication by reducing cellular lipid content.

As is well known, NSP9 is one of the components of the PEDV VRCs (24). Therefore, studying the interaction between PEDV NSP9 and cellular proteins will contribute new information to the replication mechanism of PEDV. Here, we identified HNRNPA3 as the interacting protein of NSP9 through mass spectrometry screening. HNRNP family proteins have been reported to play a critical function in the life cycle of (+) RNA viruses, such as viral genome replication and viral protein translation (36, 37). However, the research on HNRNPA3 is mainly focused on its role in disease occurrence, but there is an absence of research on its function in virus infection. Our present study found that PEDV downregulates HNRNPA3 expression *in vivo* and *in vitro*, and knocking down HNRNPA3 can promote virus replication. It is worth noting that a few studies have shown that host proteins could inhibit viral replication by affecting viral RNA synthesis (27, 38). Similarly, in our study, knocking down HNRNPA3 significantly increased the level of PEDV dsRNA and enhanced the protein levels of lipid synthesis-related genes. The use of miR-218b-5p inhibitor significantly reduced viral load, which is a very promising discovery. In chimpanzee models, miR-122 inhibitor could prevent HCV infection and replication (39). Our work also provides new insights for further research on how to apply miRNA to restrict PEDV infection *in vivo*.

Next, we further explored the mechanism by which HNRNPA3 affects SREBF1 activation. It has been reported that the activation of SREBP-1a by the hepatitis B virus HBx protein requires the participation of two bZIP transcription factors C/EBP and E4BP4 (40). Additionally, transcription factor SP1 is critical for SREBP1a activation by the CVB3 nonstructural protein 2A protein (27). We partitioned the promoter sequence of SREBF1, and the critical region of HNRNPA3 affecting SREBF1 activity was finally identified. ZNF135 is a novel transcription factor utilized by HNRNPA3 to regulate the activation of SREBF1. Previous studies have reported that cell signaling is also closely associated with SREBP1 activation, such as PI3K/Akt, MEK, and JNK cascades (26, 27). In our study, we discovered that only using PI3K and JNK inhibitors effectively affected the activation of SREBF1. Cellular lipid metabolism is a very complex process, and it is normal that different mechanisms exist during various viral infections.

**Fig 8:**
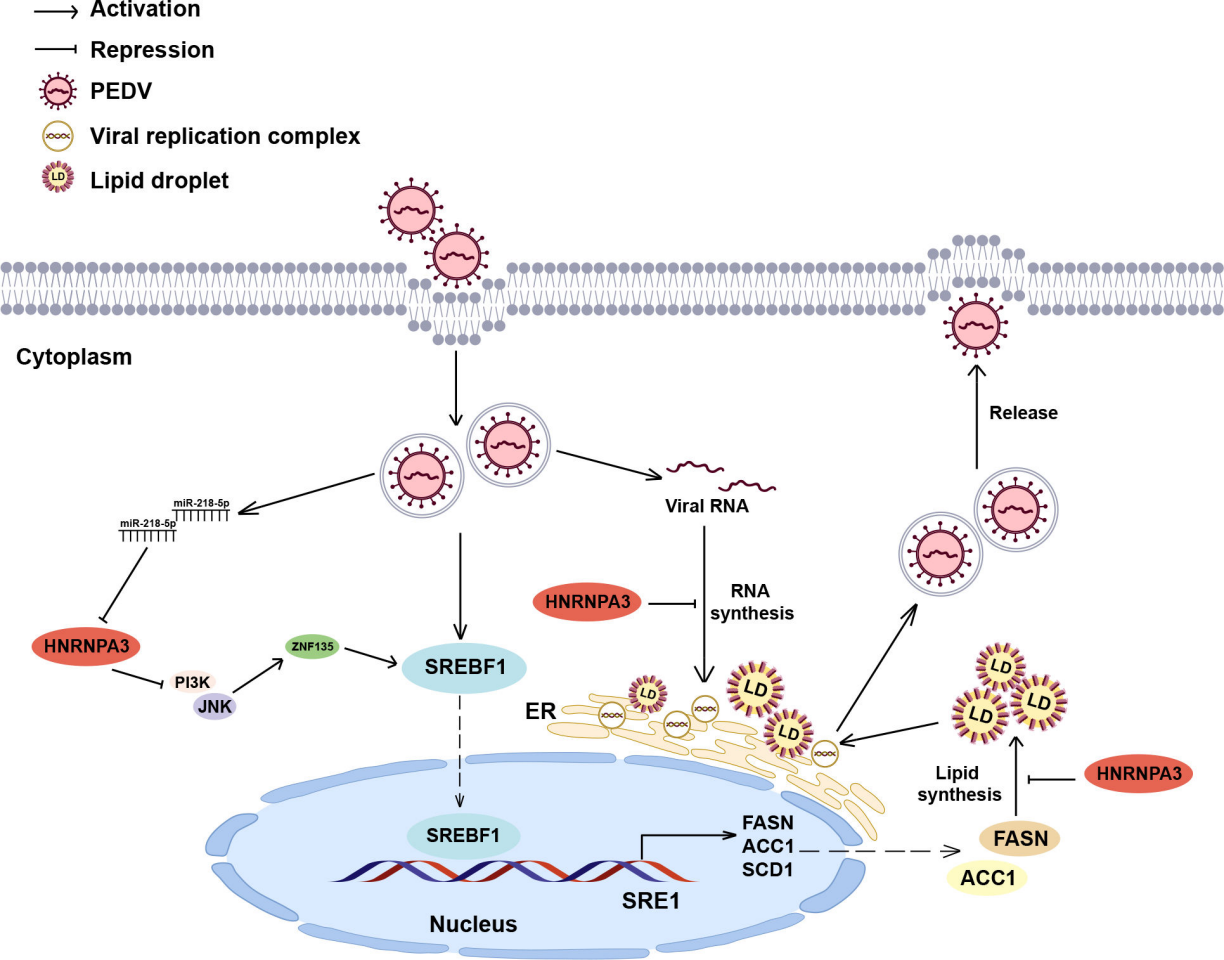
The schematic model of the role of HNRNPA3 in PEDV infection. PEDV infection promotes the expression of SREBF1 and its downstream genes, facilitating viral replication by increasing cellular lipid accumulation. HNRNPA3 is a negative regulator of SREBF1, which reduces lipid synthesis and impairs viral RNA synthesis.

In summary, we identified a novel host factor, HNRNPA3, that is critical for PEDV infection (Fig. 8). Furthermore, we found that HNRNPA3 exerts inhibitory effects on SREBF1 expression through the ZNF135 as well as PI3K/AKT and JNK signaling pathways. Our study elucidates a novel mechanism of HNRNPA3 regulating PEDV replication, which contributes to our understanding of how PEDV promotes its replication by regulating cell lipid metabolism.

## MATERIALS AND METHODS

### Cells and virus

Marc-145 and HEK293T cells were grown in Dulbecco’s modified eagle medium (DMEM HyClone) supplemented with 10% fetal bovine serum (FBS, Gibco). All media were supplemented with antibiotics (penicillin 100 U/mL, streptomycin 100 µg/mL; Solarbio) and incubated under 37°C and 5% CO_2_ conditions. The PEDV strain CH/SXYL/2016 (GenBank: MF462814.1) was isolated and maintained in our laboratory (41). The virus samples were diluted with serial 10-fold dilutions and added to the cells cultured in 96-well plates. Cells were washed twice after 1 h of adsorption and then cultured in DMEM supplemented with 2% FBS. The cytopathogenic effect was determined, and the TCID_50_ was calculated using the Reed-Muench method (42).

### Antibodies and reagents

The mouse polyclonal anti-PEDV N and anti-PEDV NSP9 antibodies were stocked in our laboratory (43). Rabbit polyclonal anti-HNRNPA3 antibody (25142-1-AP), mouse monoclonal anti-FASN antibody (66591-1-lg), rabbit polyclonal anti-SREBF1 antibody (14088–1-AP), mouse monoclonal anti-ACC antibody (67373–1-lg), and mouse monoclonal anti-β-actin (66009-1-lg) were from Proteintech. Rabbit polyclonal anti-SAPK/JNK antibody (9252) and rabbit polyclonal anti-PI3 kinase antibody (4252) were from Cell Signaling Technology. Rabbit polyclonal anti-ZNF135 antibody (NBP1-80913) was from Novus Biologicals. Mouse anti-Flag-tag antibody (HT201-01) was from TransGen Biotech. The mouse anti-GFP-tag antibody (AB0005) was from Abways. Mouse anti-double-stranded RNA (J2) (10010200) was from Scicons. Alexa Fluor 594 conjugated anti-rabbit IgG (HA1122) and Alexa Fluor 594 conjugated anti-mouse IgG (HA1126) were from HUABIO Technology. HRP-conjugated secondary antibodies were from Shanghai Diyi Biotechnology.

AM580, SP600125, Trametinib, LY294002, BODIPY493/503, and dimethyl sulfoxide were obtained from MedChemExpress. Protein A/G PLUS-Agarose (sc-2003) was obtained from Santa Cruz. Negative control (NC) siRNA, HNRNPA3-siRNA, SREBF1-siRNA, JNK-siRNA, PI3K-siRNA, miR-218-5p mimic/inhibitor, and control mimic/inhibitor were purchased from Sangon Biotech. The sequences of miRNA mimic/inhibitor and siRNAs are listed in Table S1.

### Plasmid construction

The pcDNA3.1-Flag-HNRNPA3 and pEGFP-NSP9 eukaryotic expression plasmids were constructed. The coding sequences of HNRNPA3 and NSP9 genes were amplified using cDNA as a template. The amplification products were digested with restriction enzyme (New England Biolabs) and cloned into the expression vectors using T4 DNA Ligase (EL0011, Thermo Scientific). The promoter sequence of SREBF1 was cloned into the pGL4.10 vector to construct pGL4.10-SREBF1. The 3′-UTR sequence of HNRNPA3 was obtained and cloned into the pmirGLO vector to construct pmirGLO-HNRNPA3-UTR. All constructed plasmids as described above were confirmed by sequencing. The primers are provided in Table S2.

### qPCR

Total RNA was extracted using TRNzol (DP424, Tiangen), and 2 µg of total RNA was reverse transcribed into cDNA using a FastKing RT kit (KR116, Tiangen). β-Actin and U6 were used as controls for mRNAs and miRNAs, respectively. qPCR analysis was performed using SYBR Green Fast qPCR Mix (RK21203, ABclonal), and the RNA level of each gene was analyzed by the 2^−ΔΔCT^ method. The primers used for qPCR are provided in Table S3.

### Western blot

Cells were lysed in RIPA lysis buffer (PL006, Zhonghui Hecai) supplemented with protease inhibitor (PL026) and phosphatase inhibitor (PL012-1). Identical quantities of samples were subjected to SDS-PAGE and transferred to PVDF membranes (Millipore). The membranes were blocked with 5% skim milk powder (CN7861, Coolaber) for 1 h and incubated with primary antibodies overnight at 4°C. Then, the membranes were washed and incubated with secondary antibodies (Diyi Biotechnology). Finally, signals were scanned with the SmartGel imaging system (SageCreation Biotechnology).

### Attachment and entry assay

The protocol used was published by Liu et al. (44). Briefly, Marc-145 cells were transfected with Flag-HNRNPA3 plasmids or siHNRNPA3 and then inoculated with PEDV (multiplicity of infection [MOI] = 10) for 1 h at 4°C to allow attachment. The cells were washed twice and collected to detect virus attachment by qPCR. For the PEDV entry experiment, the transfected cells were inoculated with PEDV (MOI = 10) for 1 h at 4°C, and then the cells were transferred to 37°C for 1 h after removing the unattached virus particles. The cells were collected and washed twice to detect virus entry by qPCR.

### Dual-luciferase reporter assays

Luciferase reporter vectors containing HNRNPA3 3′-UTR-WT or 3′-UTR-Mut were co-transfected with control mimic or miR-218-5p mimic in HEK293T cells. At 24 h post-transfection, the cell lysate supernatant was analyzed using the dual luciferase assay kit (Promega). The values of luciferase obtained from the assays were normalized with Renilla luciferase activity.

To detect the activation of the SREBF1 promoter, cells were transfected with the luciferase reporter vectors, phRL-TK plasmids, and HNRNPA3-siRNA or NC-siRNA. Then, cells were untreated or treated with PEDV for 24 h. Cell lysates were prepared and analyzed by a dual luciferase assay kit (Promega).

### Immunofluorescence staining

Marc-145 cells treated with different conditions were collected and immediately treated with 4% paraformaldehyde (BL539A, Biosharp) for 20 min. Cells were permeabilized in 0.1% Triton X-100 (BS084, Biosharp) for 15 min and then incubated with 5% bovine serum albumin for 1 h. After blocking nonspecific binding, cells were incubated with specific antibodies overnight at 4°C. The cells were washed and incubated with secondary antibodies for 1 h. The LDs were stained with BODIPY493/503 for 10 min, the nuclei were stained with DAPI (BL105A, Biosharp) for 10 min. Finally, all samples were analyzed using the Leica TCS SP8 fluorescence microscope (Leica, Germany).

### Animal experiment

For the PEDV infection trial, six newborn piglets were randomly divided into the PEDV-infected and the control groups. Each experimental group was housed in a separate incubator and fed 15 mL of liquid milk replacer every 3 h. Piglets in the PEDV-infected group were orally inoculated with PEDV (1.5 mL, 10^5.5^ TCID_50_), while the control group was inoculated with an equivalent amount of sterile PBS solution. At 3 dpi, piglets from each group infected with PEDV or mock-infected were euthanized, and intestinal samples were collected for immunohistochemistry and western blot analysis.

### Immunohistochemistry

The small intestine samples were embedded in paraffin and sectioned, then dewaxed in xylene, and rehydrated. For immunohistochemical staining, the sections were treated with citrate buffer (pH 6.0) and blocked with 5% normal goat serum, then incubated with primary antibody overnight at 4°C. The sections were incubated with a secondary antibody at room temperature for 1 h, treated with the DAB Horseradish Peroxidase Color Development Kit (P0202, Beyotime), and stained with hematoxylin for 30 s. Following each operation step, the sections were washed three times for 5 min in 0.1-M PBS (pH 7.2–7.4). The sections were visualized using the Nikon Ni-U microscope.

### Statistical analysis

Data are presented as the mean ± standard deviation. Statistical analysis was performed using GraphPad Prism software. The levels of significance were considered as *P < 0.05, **P < 0.01.

## SUPPLEMENTAL MATERIAL

Supplemental figures mbio.03197-23-s0001.docx Fig. S1 to S6.

Table S1 mbio.03197-23-s0002.xlsx Sequences of miRNA mimic and inhibitor and siRNAs used in this study.

Table S2 mbio.03197-23-s0003.xlsx Sequences of primers used for plasmid construction in this study.

Table S3 mbio.03197-23-s0004.xlsx Sequences of primers used for qPCR in this study.

ASM does not own the copyrights to Supplemental Material that may be linked to, or accessed through, an article. The authors have granted ASM a non-exclusive, world-wide license to publish the Supplemental Material files. Please contact the corresponding author directly for reuse.
