# Supplementary material for: PEDV inhibits HNRNPA3 expression by miR-218-5p to enhance cellular lipid accumulation and promote viral replication
Source: mBio. 2024 Jan 23;15(2):e03197-23. doi: 10.1128/mbio.03197-23 (PMC10865979; doi:10.1128/mbio.03197-23)
Supplement: Supplemental figures — Fig. S1 to S6. [file mbio.03197-23-s0001.docx]

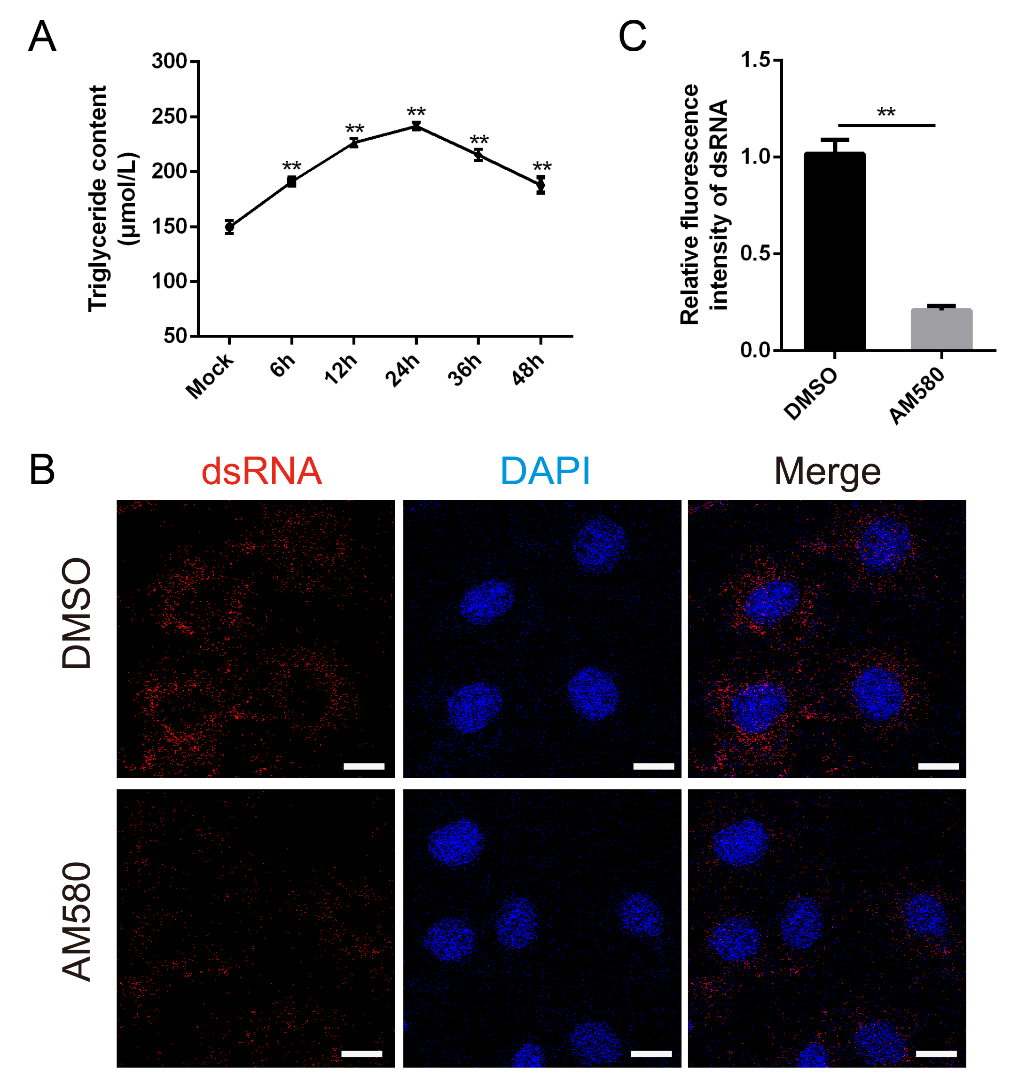


**Fig S1** The inhibitory effect of AM580 on PEDV dsRNA synthesis. (A) Marc-145 cells were infected with PEDV (MOI=1), and the triglyceride contents were detected at different time points. (B) Marc-145 cells were infected with PEDV (MOI=1) and treated with varying concentrations of AM580 (10 μM) or vehicle (DMSO, 1:1000). The content of PEDV dsRNA (red) was detected by immunofluorescence assay. Scale bar, 12 μm. The fluorescence intensity of dsRNA was analyzed with ImageJ. Three independent experiments were carried out. Error bars represent the mean ± SD for triplicate experiments, **P* < 0.05, ***P* < 0.01.


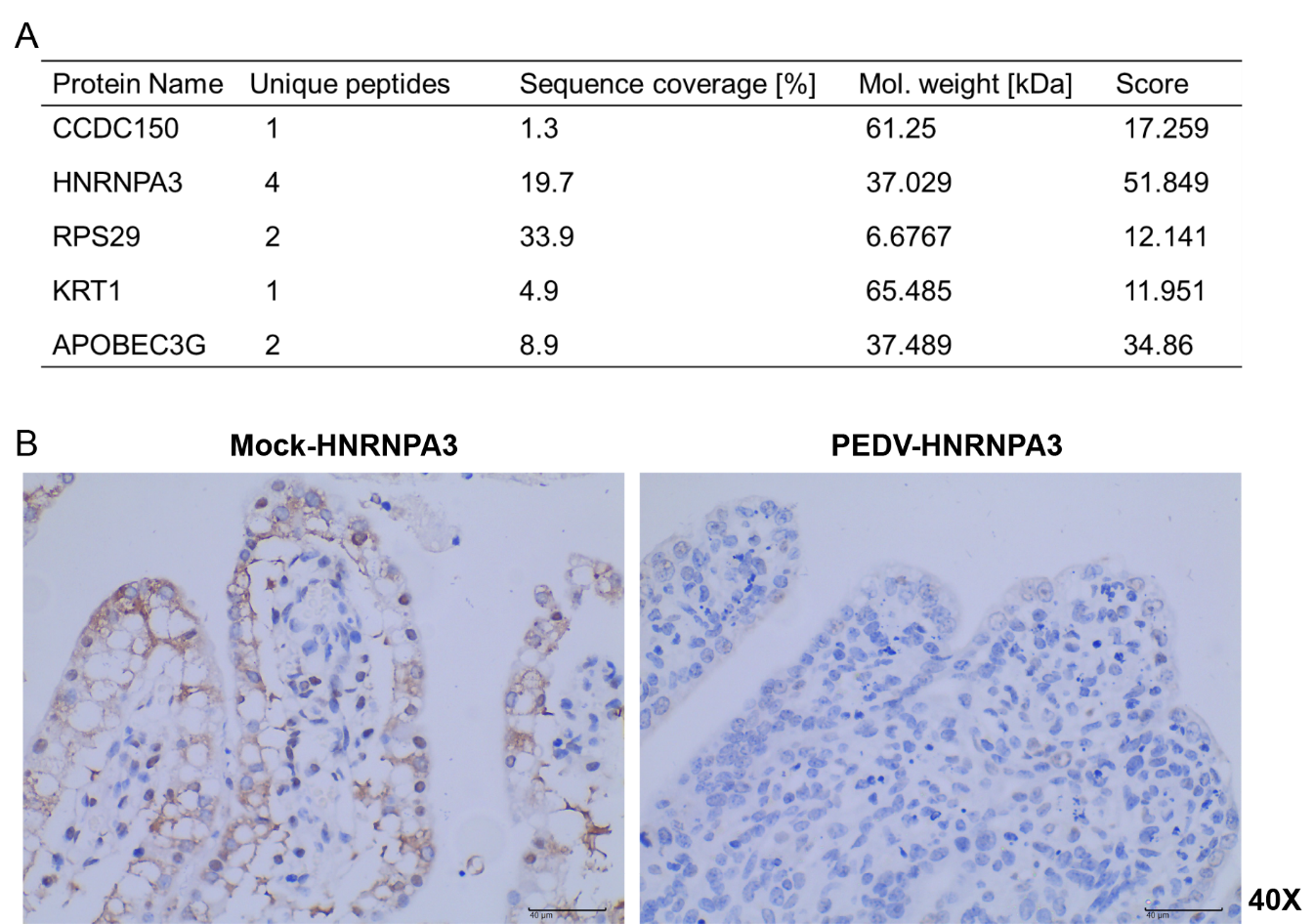


**Fig S2** Identification of PEDV NSP9-interacting proteins. (A) Identified proteins from the immunoprecipitation reaction of NSP9-transfected cell lysates. (B) Expression of HNRNPA3 in the small intestine of the mock-infected and PEDV-infected group was detected by immunohistochemistry. HNRNPA3 was stained deep yellow-brown (Magnification × 40).


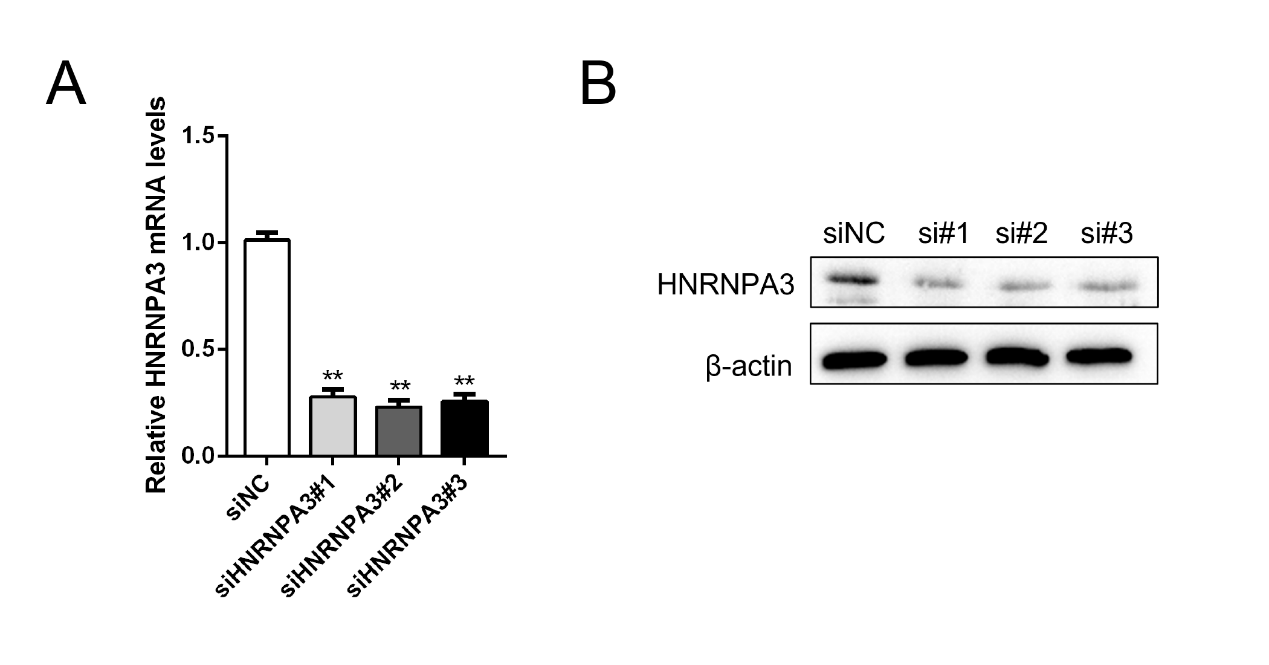


**Fig S3** The interference efficiency of HNRNPA3 siRNAs. (A and B) Marc-145 cells were transfected with siNC or HNRNPA3 siRNAs (#1, #2, and #3), and the mRNA and protein levels of HNRNPA3 were determined by qPCR and western blot. β-actin was used as a loading control. Three independent experiments were carried out. Error bars represent the mean ± SD for triplicate experiments, **P* < 0.05, ***P* < 0.01.


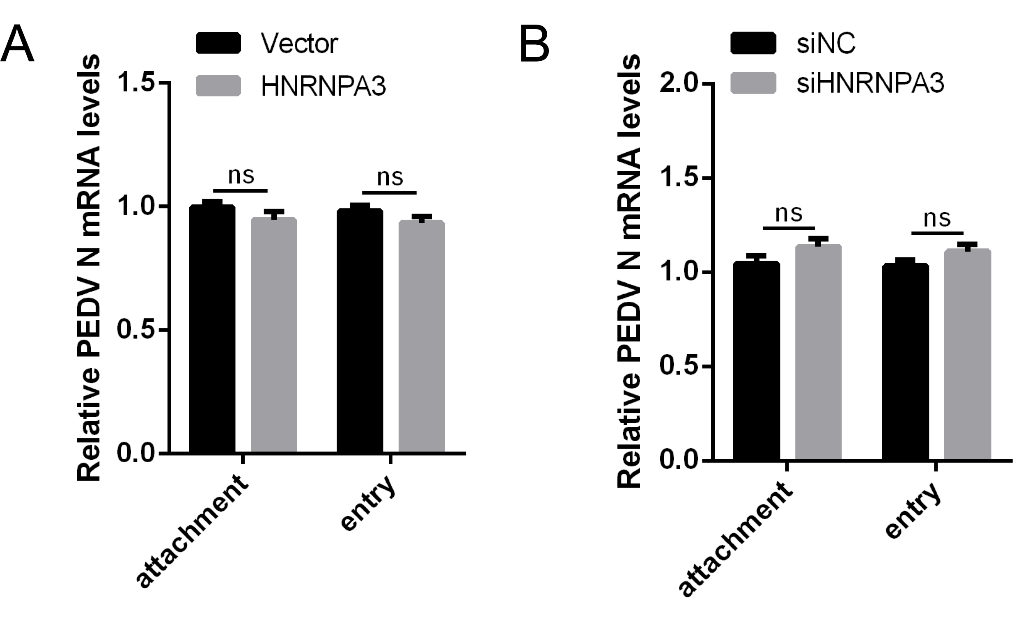


**Fig S4** The effects of HNRNPA3 on PEDV attachment and entry. (A and B) Marc-145 cells were transfected with vector or Flag-HNRNPA3 and siNC or siHNRNPA3 and infected with PEDV (MOI=10) for 1 h at 4°C and then at 37°C at 0 hpi (attachment) or 1 hpi (entry). The unbound virus was removed, and the mRNA levels of PEDV-N were detected by qPCR.


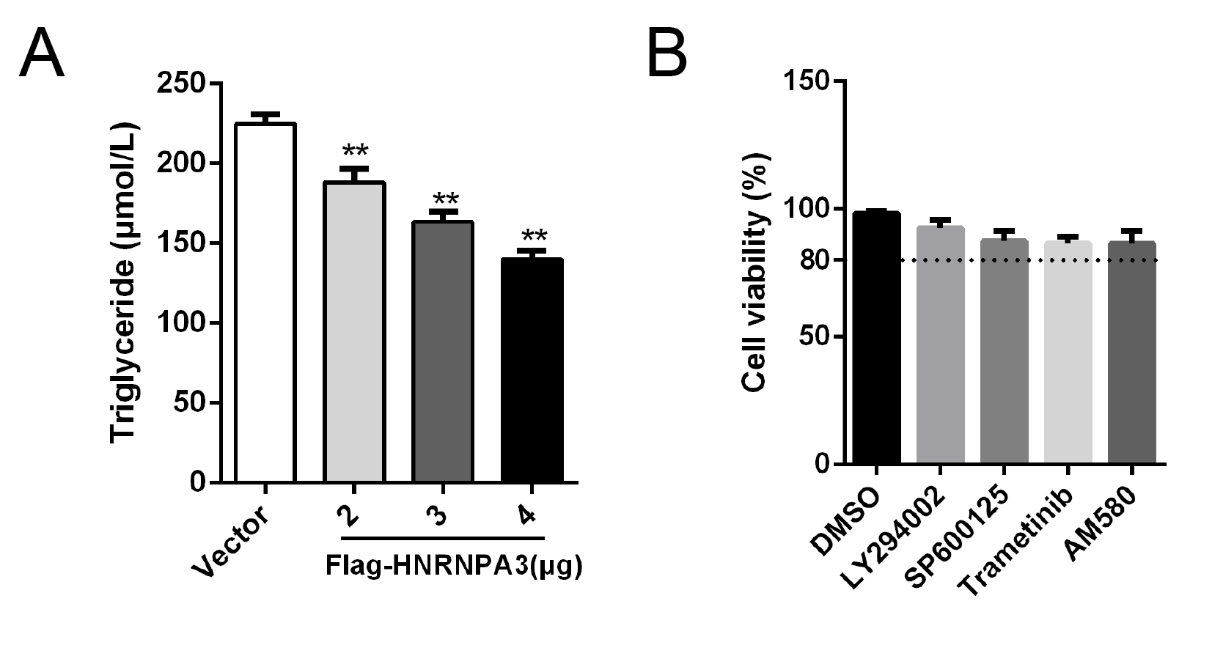


**Fig S5** Quantification of triglycerides in Flag-HNRNPA3 transfected cells. (A) Marc-145 cells were transfected with vectors or increasing amounts of the Flag-HNRNPA3 plasmids, and the triglyceride contents were detected. (B) Marc-145 cells were grown in 96-well plates and incubated with different inhibitors used in the study (10 µM LY294002, 10 µM SP600125, 10 µM Trametinib, 10 µM AM580, and DMSO) for 24 h and MTT assays were performed. Three independent experiments were carried out. Error bars represent the mean ± SD for triplicate experiments, **P* < 0.05, ***P* < 0.01.


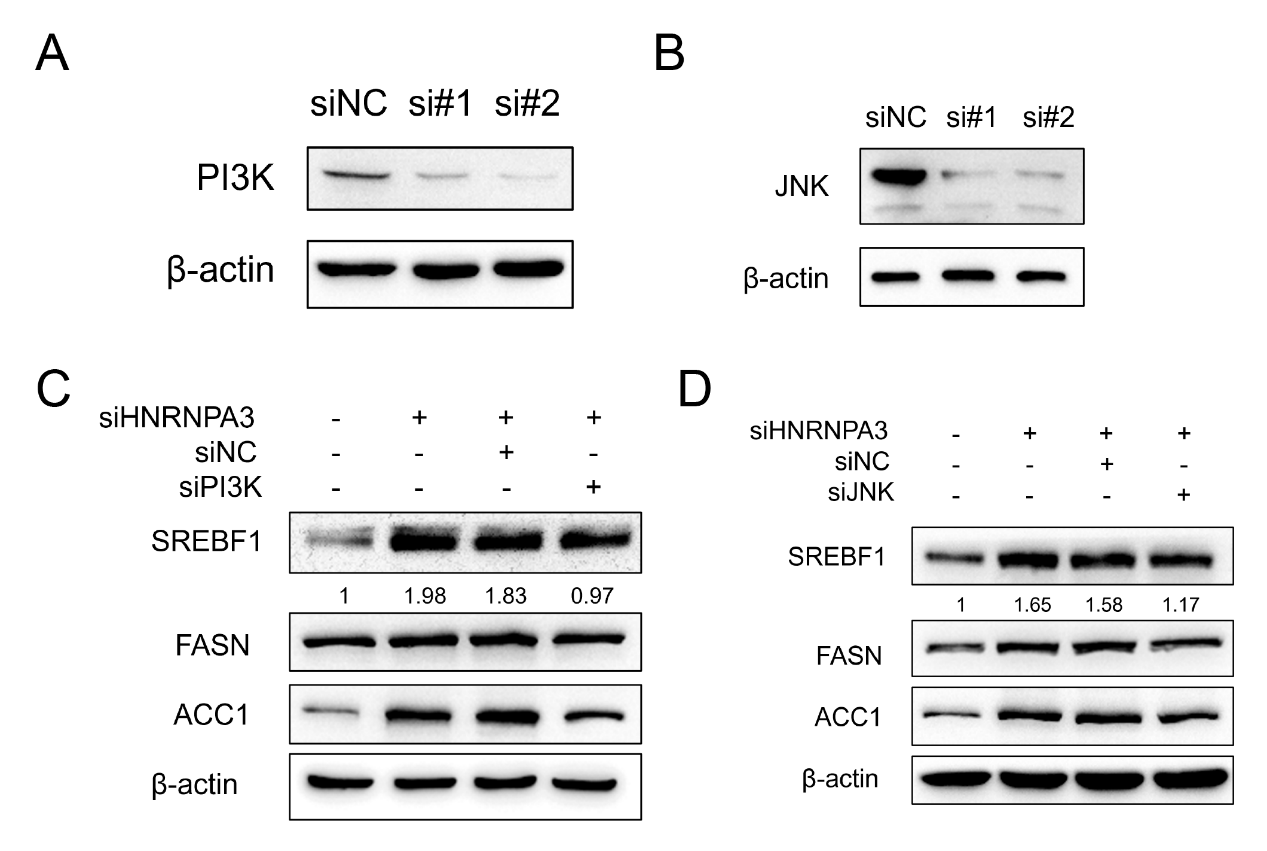


**Fig S6** Role of the PI3K/AKT and JNK pathways in SREBF1 activation by knocking down HNRNPA3. (A and B) Marc-145 cells were transfected with siNC or PI3K/JNK siRNAs (#1 and #2), and the protein levels were determined by western blot. β-actin was used as a loading control. (C and D) Marc-145 cells were transfected with siHNRNPA3 and siPI3K or siJNK and infected with PEDV (MOI=0.5). The protein levels of SREBF1 were determined by western blot. β-actin was used as a loading control. Three independent experiments were carried out. The intensities of bands were quantified by ImageJ.
